# Investigating the relationship between breast cancer risk factors and an AI-generated mammographic texture feature in the Nurses’ Health Study II

**DOI:** 10.1038/s41523-025-00870-4

**Published:** 2025-12-23

**Authors:** Xueyao Wu, Shu Jiang, Aaron Ge, Constance Turman, Graham Colditz, Rulla M. Tamimi, Peter Kraft

**Affiliations:** 1 Division of Cancer Epidemiology and Genetics, National Cancer Institute, Rockville, MD USA; 2 Washington University School of Medicine in St. Louis, St. Louis, MO USA; 3 University of Maryland School of Medicine, Baltimore, MD USA; 4 Program in Genetic Epidemiology and Statistical Genetics, Harvard T.H. Chan School of Public Health, Boston, MA USA; 5 Population Health Sciences Department, Weill Cornell Medical School, New York, NY USA; 6 Channing Division of Network Medicine, Department of Medicine, Brigham and Women’s Hospital and Harvard Medical School, Boston, MA USA

**Keywords:** Risk factors, Breast cancer, Cancer epidemiology, Cancer genetics, Cancer imaging

## Abstract

The mammogram risk score (MRS), an AI-driven mammographic texture feature, strongly predicts breast cancer risk independently of breast density, though underlying mechanisms remain unclear. Using data from the Nurses’ Health Study II (292 cases, 561 controls), we validated MRS’s association with breast cancer and evaluated its relationships with established breast cancer risk factors through observational analyses, polygenic score analyses, and Mendelian randomization. MRS was significantly associated with breast cancer risk before (OR=1.92 per SD increase; 95% CI:1.57 to 2.35; 10-year AUC=0.69) and after adjustment for predicted BI-RADS density (OR=1.85; 95% CI:1.49 to 2.30). Early life body size and adult body mass index (BMI) were inversely associated with MRS, while benign breast disease history and predicted BI-RADS density showed positive associations; after adjusting for density, associations between MRS and the other three risk factors were attenuated. Polygenic score analyses and Mendelian randomization consistently demonstrated significant positive associations between genetic predictors of breast density measures (dense area, percent density, predicted BI-RADS density) and MRS. After adjusting for predicted BI-RADS density and BMI, genetic predictors of higher waist-to-hip ratio were significantly associated with increased MRS. Our findings reveal robust associations between breast density measures and MRS and suggest a potential impact of central obesity on MRS. Future larger-scale validation studies are needed.

## Introduction

Breast cancer remains the most prevalent malignant cancer among women worldwide^1^. While advances in mammographic screening have facilitated early detection and risk stratification by assessing breast density^2^, traditional measures of mammographic density primarily evaluate the relative amounts of fibroglandular tissue (i.e., the functional breast tissue composed of epithelial and stromal cells). This approach limits our ability to fully capture the heterogeneity of individual breast tissue features, such as architecture and spatial relations^3^. Recent research has leveraged accumulating digital mammogram datasets coupled with sophisticated computational techniques to quantify texture features of mammograms, aiming for more precise and individualized risk predictions. These texture features capture detailed patterns and variations in breast tissue that go beyond simple density measurements^4^. A notable development in this area is the mammogram risk score (MRS), an innovative, artificial intelligence (AI)-driven texture feature derived from whole mammogram images that robustly predicts breast cancer risk independently of breast density (5-year area under the receiver operating characteristic curve [AUC] = 0.75)^5,6^. However, given that the MRS is derived from a deep learning model that lacks inherent interpretability, its biological underpinnings remain unclear.

The risk of breast cancer is influenced by multiple factors beyond age and genetic markers. Lifestyle, behavioral, and developmental factors, such as anthropometric measures and reproductive events, collectively contribute to breast cancer susceptibility^7^ and may also relate to features in breast tissue. Epidemiological studies have highlighted significant associations between traditional measures of mammographic density and various risk factors, including early life and adult adiposity^8–11^, height^12,13^, age at menarche^12,13^, age at first birth^14,15^, age at natural menopause^16^, and other reproductive/hormonal factors^17^. Utilizing germline genetic variants as instrumental variables (IVs) to strengthen causal inference, Mendelian randomization (MR) studies have reinforced associations with early life and adult adiposity^18,19^, offering protection against confounding and reverse causation typical in observational studies^20^.

Given that texture features capture distinct aspects of breast tissue from summary density measures, investigating how established risk factors relate to these features could improve our understanding of their underlying biology and provide valuable insights into breast cancer pathogenesis. Previous studies have demonstrated phenotypic and genetic relationships between adiposity and V^21,22^, a texture feature reflecting grayscale intensity variations on digitized film mammograms^23^. MRS, by comparison, was developed using supervised machine learning to not only predict variation in whole digital images more accurately but also to capture biological features relevant to breast cancer risk^6^. These characteristics make MRS a promising target for investigation aimed at advancing breast cancer prevention. Yet, to date, no observational or MR study has explored these associations for the MRS.

With an overarching goal of deepening the understanding of the biological underpinnings of MRS and its potential role in breast cancer susceptibility, the present study comprehensively investigates the relationships between established breast cancer risk factors—encompassing anthropometrics, reproductive and hormonal factors, family history, and traditional mammographic density metrics—and MRS, through comprehensive observational and genetic analyses performed within the Nurses’ Health Study II (NHS II).

## Results

### Participant characteristics and MRS-breast cancer association in the NHS II

This nested case-control study comprised 853 women (292 cases and 561 controls) with a mean age of 55.3 (±5.45 years) at the time of the mammogram. The majority (64.1%) were postmenopausal. A comparison of risk factor distribution between groups divided by median MRS can be found in Table 1. Notably, compared to those below the median, participants with above-median MRS were younger, more likely to have lower body mass index (BMI) and waist-to-hip ratio (WHR), denser breasts, a history of benign breast disease at the time of mammogram, and more likely to be breast cancer cases (47.8% vs 20.7%) (all *P* < 0.05).

**Table 1:** Baseline characteristics of participants according to median mammogram risk score

Characteristic	All	MRS < the median level	MRS ≥ the median level	*P*
	*N* = 853	*N* = 426	*N* = 427	
Age, years	55.3 (5.45)	56.4 (5.21)	54.3 (5.48)	<0.001
Early life body size	2.56 (1.16)	2.64 (1.21)	2.49 (1.10)	0.059
Body mass index, kg/m^2^	26.8 (6.06)	27.4 (6.45)	26.2 (5.57)	0.003
Waist-to-hip ratio	0.83 (0.07)	0.83 (0.07)	0.82 (0.07)	0.035
Waist-to-hip ratio adjusted for BMI	0.00 (0.07)	0.00 (0.07)	0.00 (0.07)	0.648
Height, inch	64.9 (2.52)	64.9 (2.48)	65.0 (2.55)	0.523
Age at menarche, years	13.2 (2.95)	13.2 (2.98)	13.2 (2.92)	0.913
Age at first birth, years	26.3 (4.93)	26.0 (4.71)	26.7 (5.12)	0.075
Menopausal status				<0.001
*Premenopausal*	233 (27.8%)	86 (20.6%)	147 (34.8%)	
*Postmenopausal*	538 (64.1%)	302 (72.4%)	236 (55.9%)	
*Unsure*	68 (8.10%)	29 (6.95%)	39 (9.24%)	
Age at natural menopause, years	49.7 (4.14)	49.5 (4.37)	50.0 (3.80)	0.094
Postmenopausal hormone use				0.108
*Premenopausal or postmenopausal not on therapy*	626 (77.4%)	322 (79.3%)	304 (75.4%)	
*Postmenopausal on therapy*	183 (22.6%)	84 (20.7%)	99 (24.6%)	
No. pregnancies ≥6 months	1.94 (1.24)	1.96 (1.25)	1.92 (1.22)	0.664
History of benign breast disease				0.013
*Yes*	525 (61.5%)	244 (57.3%)	281 (65.8%)	
*No*	328 (38.5%)	182 (42.7%)	146 (34.2%)	
Predicted BI-RADS density^a^				<0.001
*a, almost entirely fatty*	40 (5.10%)	33 (8.44%)	7 (1.78%)	
*b, scattered areas of fibroglandular tissue*	320 (40.8%)	209 (53.5%)	111 (28.2%)	
*c, heterogeneously dense*	396 (50.4%)	140 (35.8%)	256 (65.0%)	
*d, extremely dense*	29 (3.69%)	9 (2.30%)	20 (5.08%)	
Family history of breast cancer				0.14
*Yes*	160 (18.8%)	71 (16.7%)	89 (20.8%)	
*No*	693 (81.2%)	355 (83.3%)	338 (79.2%)	
Breast cancer cases				<0.001
*Yes*	292 (34.2%)	88 (20.7%)	204 (47.8%)	
*No*	561 (65.8%)	338 (79.3%)	223 (52.2%)	
Mammogram risk score	0.00 (1.00)	−0.84 (0.58)	0.84 (0.49)	<0.001

^a^Predicted BI-RADS density was assessed using a deep learning algorithm previously developed to predict mammographic breast density from digital mammograms. The algorithm categorizes breasts from a (almost entirely fatty) to d (extremely dense), matching an experienced mammographer’s evaluation.

External validation in NHS II, which included 201 cases and 561 controls after excluding cases diagnosed within 6 months of mammography, demonstrated a strong association between MRS and breast cancer risk (odds ratio [OR] = 1.92 per standard deviation [SD] difference in MRS; 95% confidence intervals [CI]: 1.57 to 2.35; *P* = 1.98 × 10^−18^; 10-year AUC = 0.69) (Supplementary Table 1, Supplementary Figs. 1 and 2). Cases were diagnosed 0.5–10.1 years (median 2.6) after the mammogram used for MRS calculation. The association remained robust after adjusting for predicted Breast Imaging Reporting and Data System (BI-RADS) density (OR = 1.85; 95% CI: 1.49 to 2.30; *P* = 2.50 × 10^−8^) (Supplementary Table 1).

### Associations between observed breast cancer risk factors and MRS

Both predicted BI-RADS density (*β* = 0.31 SD difference in MRS per SD difference in predicted BI-RADS density; 95% CI: 0.25 to 0.38; *P* = 1.94 × 10^−20^) and history of benign breast disease (*β* = 0.23 SD difference in MRS with vs. without history; 95% CI: 0.10 to 0.36; *P* = 4.50 × 10^−4^) showed positive associations with MRS. Early life body size (*β* = −0.08 SD difference in MRS per SD difference in body size; 95% CI: −0.14 to −0.02; *P* = 9.59 × 10^−3^) and adult BMI (*β* = −0.08 SD difference in MRS per SD difference in BMI; 95% CI: −0.14 to −0.02; *P* = 1.11 × 10^−2^) demonstrated negative associations. No statistically significant associations were observed for the other examined risk factors (all *P* > 0.05, Table 2). These results remained consistent in both direction and statistical significance when restricted to controls only or additionally adjusted for menopausal status (Supplementary Tables 2 and 3). When adjusted for predicted BI-RADS density, associations with history of benign breast disease (*β* = 0.11), early life body size (*β* = −0.02), and adult BMI (*β* = 0.05) were all attenuated towards the null (Table 2, Supplementary Table 2).

**Table 2:** Linear regression of mammogram risk score on each breast cancer risk factor

Risk factor^a^	Crude model^b^				Model adjusted for predicted BI-RADS density^c^			
	*N*	BETA	SE	*P*	*N*	BETA	SE	*P*
Early life body size	837	−0.082	0.032	9.59×10^−^^3^	770	−0.022	0.032	0.479
Body mass index	849	−0.082	0.032	1.11×10^−2^	781	0.049	0.035	0.165
Waist-to-hip ratio	728	−0.059	0.034	0.085	668	−0.006	0.035	0.868
Waist-to-hip ratio adjusted for BMI	725	−0.006	0.034	0.869	665	0.008	0.034	0.807
Height	853	−0.024	0.032	0.440	785	−0.011	0.031	0.720
Age at menarche	850	0.003	0.032	0.922	782	−0.016	0.032	0.619
Age at first birth	699	0.031	0.035	0.384	649	0.037	0.034	0.277
Age at natural menopause^d^	535	0.075	0.042	0.074	489	0.059	0.041	0.152
Postmenopausal hormone use^d^	518	0.139	0.094	0.139	475	0.022	0.093	0.815
No. pregnancies ≥6 months	761	−0.005	0.033	0.876	722	0.029	0.033	0.387
History of benign breast disease	853	0.229	0.065	4.50 × 10^−4^	785	0.111	0.065	0.091
Predicted BI-RADS density	785	0.314	0.033	1.94 × 10^−20^	-	-	-	-
Family history of breast cancer	853	0.017	0.081	0.838	785	0.003	0.080	0.974

*BMI* body mass index.

^a^All risk factors reflect measurements taken at adulthood, either at cohort baseline or at the time of the mammogram, except for early life body size, which reflects recalled values from childhood.

^b^Models include adjustments for age and breast cancer case-control status.

^c^Models include adjustments for age, predicted BI-RADS density, and breast cancer case-control status.

^d^Analyses of age at natural menopause and postmenopausal hormone use were limited to postmenopausal women.

### Associations between polygenic scores for breast cancer risk factors and MRS

Linear regressions of MRS on polygenic score (PGS) for risk factors revealed significant positive associations for dense area (*β* = 0.16 SD difference in MRS per SD difference in PGS; 95% CI: 0.06 to 0.25; *P* = 1.37 × 10^−3^) and percent density (*β* = 0.14 SD difference in MRS per SD difference in PGS; 95% CI: 0.05 to 0.23; *P* = 3.29 × 10^−3^). No significant associations were observed between the PGS for other risk factors and MRS (Table 3). A similar pattern of associations was observed in analyses restricted to controls and in models adjusted for menopausal status (Supplementary Tables 4 and 5). After adjusting for predicted BI-RADS density, the association for percent density remained strong, whereas the association for dense area weakened slightly. A significant association was additionally observed between higher PGS for WHR adjusted for BMI (WHRadjBMI) and increased MRS (*β* = 0.12 SD difference in MRS per SD difference in PGS; 95% CI: 0.03 to 0.21; *P* = 1.18 × 10^−2^) (Table 3, Supplementary Table 4).

**Table 3:** Linear regression of mammogram risk score on polygenic score for each breast cancer risk factor

Risk factor^a^	Crude model^b^				Model adjusted for predicted BI-RADS density^c^			
	*N*	BETA	SE	*P*	*N*	BETA	SE	*P*
Early life body size	383	-0.027	0.050	0.588	353	0.035	0.050	0.482
Adult body size	383	-0.003	0.049	0.950	353	0.037	0.049	0.444
Waist-to-hip ratio	383	0.008	0.048	0.870	353	0.073	0.048	0.124
Waist-to-hip ratio adjusted for BMI	383	0.071	0.048	0.139	353	0.118	0.047	1.18 × 10^−02^
Height	383	-0.004	0.049	0.932	353	-0.027	0.048	0.580
Age at menarche	383	-0.022	0.049	0.658	353	-0.059	0.049	0.223
Age at first birth	383	-0.006	0.049	0.908	353	-0.033	0.047	0.489
Age at natural menopause	383	-0.034	0.049	0.492	353	-0.045	0.047	0.344
Number of children ever born	383	-0.055	0.049	0.261	353	-0.044	0.048	0.357
Dense area	383	0.155	0.048	1.37 × 10^−03^	353	0.093	0.049	0.062
Non-dense area	383	0.001	0.048	0.979	353	0.009	0.047	0.850
Percent density	383	0.141	0.048	3.29 × 10^−03^	353	0.126	0.048	9.36 × 10^−03^

*BMI* body mass index.

^a^All risk factors reflect measurements taken at adulthood, except for early life body size, which reflects recalled values from childhood.

^b^Models include adjustments for age, genotyping platform, the top 10 genetic principal components, and breast cancer case-control status.

^c^Models include adjustments for age, predicted BI-RADS density, genotyping platform, the top 10 genetic principal components, and breast cancer case-control status.

### Mendelian randomization between breast cancer risk factors and MRS

Linear regressions of risk factors on their corresponding PGS revealed significant genetic associations for 7 risk factors, including height, BMI, age at menarche, early life body size, WHR, predicted BI-RADS density, and age at natural menopause (*F*-statistics: 212.56 to 5.36, Supplementary Table 6). While PGS for dense area was significantly associated with predicted BI-RADS density (*R*^2^ = 0.03, *F* = 9.09, *P* = 2.76 × 10^−3^), PGS for percent density showed no association (*R*^2^ = 0.00, *F* = 1.47, *P* = 0.23). The dense area PGS was thus used as an IV for predicted BI-RADS density in subsequent two-stage least squares (2SLS) analyses. WHRadjBMI, age at first birth, and number of children ever born were excluded from 2SLS due to weak instrument strength (*F*-statistic < 5). Associations adjusted for menopausal status or predicted BI-RADS density are detailed in Supplementary Tables 7 and 8.

2SLS analyses found a significant association between genetically predicted BI-RADS density and MRS (*β* = 0.84 SD difference in MRS per SD difference in predicted BI-RADS density; 95% CI: 0.21 to 1.46; *P* = 8.85 × 10^−3^), while identifying no statistically significant associations between the other 6 genetically predicted risk factors and MRS. Among the other risk factors, the strongest effect estimates were observed for age at natural menopause (*β* = −0.80 SD difference in MRS per SD difference in genetically predicted age at natural menopause, 95% CI: −2.58 to 0.98, *P* = 0.38) and early life body size (*β* = −0.13 SD difference in MRS per SD difference in genetically predicted early life body size, 95% CI: −0.59 to 0.33, *P* = 0.57), neither of which reached statistical significance (Table 4). All additional adjustments yielded similar results (Table 4, Supplementary Tables 9 and 10).

**Table 4:** Two-stage least squares regression between each breast cancer risk factor (exposure) and mammogram risk score (outcome)

Risk factor^a^	Crude model^b^				Model adjusted for predicted BI-RADS density^c^			
	*N*	BETA	SE	*P*	*N*	BETA	SE	*P*
Early life body size	370	−0.132	0.234	0.574	341	0.215	0.269	0.424
Body mass index	381	−0.012	0.175	0.946	351	0.153	0.201	0.446
Waist-to-hip ratio	336	0.081	0.304	0.790	307	0.702	0.542	0.196
Height	383	−0.007	0.084	0.932	353	−0.045	0.080	0.579
Age at menarche	383	−0.081	0.183	0.659	353	−0.222	0.186	0.232
Age at natural menopause^d^	259	−0.800	0.907	0.378	240	−1.068	1.388	0.442
Predicted BI-RADS density	353	0.835	0.317	8.85 × 10^−03^	-	-	-	-

^a^All risk factors reflect measurements taken at adulthood, except for early life body size, which reflects recalled values from childhood.

^b^Models include adjustments for age, genotyping platform, the top 10 genetic principal components, and breast cancer case-control status.

^c^Models include adjustments for age, predicted BI-RADS density, genotyping platform, the top 10 genetic principal components, and breast cancer case-control status.

^d^Analyses of age at natural menopause were restricted to postmenopausal women.

**Fig. 1:**
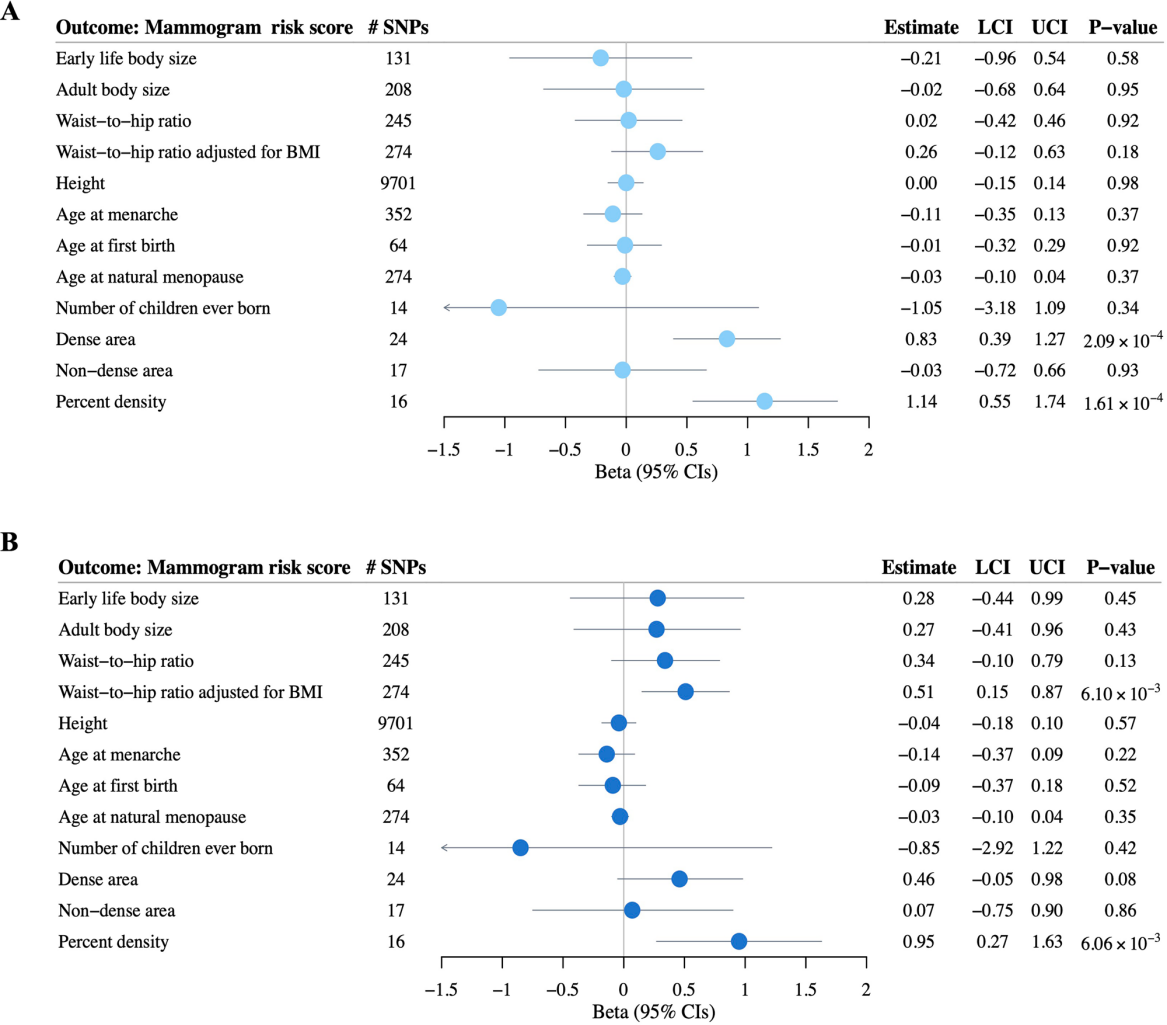
Two-sample Mendelian randomization analysis examining associations between genetically predicted risk factors (exposures) and mammogram risk score (outcome). Results are shown **A** before and **B** after adjusting for predicted BI-RADS density in Nurses’ Health Study II (NHS II). Point estimates (circles) and 95% confidence intervals (error bars) were calculated using the inverse-variance weighted approach. Effect estimates are interpreted as the standard deviation change in MRS per one-unit increase in the exposure as defined in the original GWAS. All risk factors represent adult measurements, except for early life body size, which was retrospectively reported for childhood. BMI body mass index, LCI lower confidence interval, UCI upper confidence interval.

Two-sample MR analyses identified significant associations between genetically predicted dense area and MRS (*β* = 0.83 SD difference in MRS per SD difference in dense area; 95% CI: 0.39 to 1.27; *P* = 2.09 × 10^−4^), and genetically predicted percent density and MRS (*β* = 1.14 SD difference in MRS per SD difference in percent density; 95% CI: 0.55 to 1.74; *P* = 1.61 × 10^−4^). No evidence supported significant causal associations with other risk factors (Fig. 1). Sensitivity analyses using MR-Egger regression, weighted median, weighted mode, and inverse-variance weighted (IVW) excluding outlier SNPs yielded consistent results (Supplementary Table 11). MR-Clust analysis found all variants for dense area and percent density clustered into a single group with similar causal effects, suggesting no evidence of heterogeneous causal mechanisms; for other risk factors, no variants showed significant effects (Supplementary Fig. 3). Chi-square tests on Wald ratios across all IVs for each risk factor revealed no statistically significant associations between risk factor-associated genetic variants and MRS (all *P* > 0.05, Supplementary Table 12).

The patterns of associations were robust to both restriction to control subjects (Supplementary Table 11) and adjustment for menopausal status (Supplementary Tables 13 and 14, Supplementary Fig. 4). Utilizing IV-outcome associations adjusting for predicted BI-RADS density, two-sample MR showed a significant association between genetically predicted WHRadjBMI and MRS (IVW: *β* = 0.51 SD difference in MRS per SD difference in WHRadjBMI; 95% CI: 0.15 to 0.87; *P* = 6.10 × 10^−3^) (Fig. 1), consistent across all sensitivity analyses. Association with percent density remained substantially unchanged, while association for genetically predicted dense area was attenuated (Fig. 1, Supplementary Tables 15 and 16, Supplementary Fig. 5).

## Discussion

To the best of our knowledge, this study presents one of the first and most comprehensive examinations to date of the relationships between known breast cancer risk factors and MRS—an AI-generated mammographic texture feature derived from full-field digital mammograms. Our analyses revealed robust phenotypic and genetic associations between various mammographic density measures—including predicted BI-RADS density, absolute dense area, and percent density—and the MRS, as well as a suggestive association between higher WHRadjBMI and increasing MRS.

Our external validation of the MRS in the predominantly White NHS II cohort demonstrated its robust predictive capability for breast cancer risk, supporting the generalizability of the algorithm to an independent population. The MRS maintained good discriminatory power for long-term risk, achieving a 10-year AUC of 0.69, which compares favorably with the 5-year AUCs reported in the original validation cohorts (0.75 in the Joanne Knight Breast Health Cohort at Washington University [WashU cohort]; 0.74 in the Emory Breast Imaging Dataset [EMBED]; 27–46% Non-Hispanic Black women)^6^. This result underscores the stability of the MRS as a risk marker over an extended follow-up period. Notably, our mutual adjustment analyses provide insight into the relationship between MRS, breast density, and breast cancer risk. The association between MRS and breast cancer incidence remained strong after adjusting for predicted BI-RADS density; conversely, the association for predicted BI-RADS density was substantially attenuated after adjusting for MRS. This pattern suggests that while both are important risk factors, MRS may capture mammographic information that is more proximally located on the causal pathway to breast cancer than summary density measures alone. These findings collectively underscore MRS’s potential to enhance breast cancer risk stratification across diverse clinical settings and populations.

Our findings align with previously reported significant phenotypic relationships between mammographic density and breast texture features^17,21,24–28^. The moderate correlation between predicted BI-RADS density and MRS (*r* ~ 0.31) further corroborates that while these measures are related, they are likely to reflect distinct aspects of mammographic information. Beyond the phenotypic association, our genetic analyses provide converging evidence for a shared genetic architecture and potential causal link between mammographic density and MRS. These findings corroborate and extend previous evidence from different texture measures and study designs that demonstrated similar causal relationships^29^, enhancing the credibility of MRS as a biologically plausible risk factor for breast cancer. Future studies should aim to elucidate the specific biological processes reflected by MRS and their implications for breast cancer etiology.

Moving beyond density, investigating the effects of lifestyle, behavioral, and developmental/biological risk factors on breast tissue characteristics, as summarized in mammograms, is crucial for extracting biological insights into modifiable factors for prevention studies and understanding pathways for potential preventive drug targets. While MRS itself represents a novel feature with limited existing literature, it is important to contextualize our findings within the existing body of research on other mammographic features. For instance, previous studies have demonstrated associations between various breast cancer risk factors and mammographic density, and between risk factors and other texture features such as V^21,22^. Our study of MRS builds upon previous findings by focusing on MRS—a supervised, risk-optimized score trained via a ResNet-18 convolutional neural network and validated in large, diverse cohorts to identify tissue patterns most predictive of future breast cancer incidence. This approach offers a complementary perspective for investigating the biology of risk-relevant mammographic changes.

The emergence of a statistically significant association between genetic predictors of WHRadjBMI and MRS only after adjusting for predicted BI-RADS density suggests that fat distribution, independent of overall body mass, might influence breast tissue characteristics in ways not fully captured by mammographic density alone. The ability of MRS to reveal this relationship indicates its value as an advanced imaging feature in reflecting nuanced aspects of breast tissue composition that may be relevant to cancer risk assessment. Future studies are needed to validate our results and investigate the biological mechanisms underlying the complex interplay between fat distribution, breast tissue texture features, and breast cancer susceptibility.

Several limitations of our study should be acknowledged. First, our sample size was relatively limited, which may have led to insufficient statistical power to detect associations with some risk factors, particularly those with smaller effect sizes. We emphasize that null findings observed should not be interpreted as definitive evidence of no association, and that larger studies with greater statistical power are needed. Second, our analysis was limited by the availability of only craniocaudal (CC)-view mammograms. As the MRS algorithm is optimized using four views, our reported predictive accuracy likely represents a conservative estimate of its full potential. Nevertheless, the strong performance achieved with this two-view approach underscores the algorithm’s utility in common, real-world scenarios where imaging sets may be incomplete, thus broadening its applicability in diverse data settings. Third, our analysis was based on a nested case-control design, which could potentially introduce ascertainment bias. However, we expect that this design would not substantially affect our results, given our careful adjustment for case-control status and the consistency of our findings in control-only analyses^30^. Fourth, while we evaluated predicted BI-RADS density (which mimics qualitative visual assessments by radiologists)^31^, we were unable to adjust for quantitative density measures due to data unavailability. This limitation may have reduced our statistical power to detect associations, potentially underestimating relationships between other breast cancer risk factors and MRS. Additionally, our 2SLS analyses may have been affected by phenotype mismatches between the traits used to derive PGS from genome-wide association studies (GWAS) and the corresponding phenotypes measured in our study. For example, the use of quantitative dense area PGS to instrument qualitative BI-RADS density categories could potentially violate the restriction exclusion assumption and introduce bias in our causal estimates.

Our study has several notable strengths. A key advantage is the availability of genetic data, digital mammogram data, and comprehensive covariate data on the same set of samples, allowing for integrated analyses across multiple domains. Triangulating evidence from both observational and genetic studies mitigated biases inherent in each study design, providing a multi-perspective evaluation of associations. The MRS algorithm was developed independently of the NHS II cohort, reducing the possibility for overfitting or circular reasoning in our analyses. Developed using standard digital mammograms, sophisticated statistical methods, and large-scale populations, the MRS itself represents an advanced texture feature with significant potential in clinical settings.

To conclude, this study provides initial insights into the etiologic underpinnings of MRS. We validated that MRS serves as a robust predictor of breast cancer risk, providing information independent of and beyond that captured by traditional density measurements. Our investigation further revealed robust associations between breast density measures and MRS and suggests a potential impact of central obesity on MRS. Future research should encompass larger-scale studies to definitively characterize these associations and elucidate the underlying biological mechanisms. As our understanding of mammographic texture features advances, tools like MRS that offer a more nuanced view of risk beyond conventional measures may become integral to personalized breast cancer assessment and prevention strategies.

## Methods

### Study participants

The current study leverages resources from the NHS II, a large prospective cohort established in 1989 with 116,429 female and predominantly White (>90%) registered nurses aged 25–42 from 14 states^32^. Between 1996 and 1999, blood samples were collected from 29,611 women, forming a blood subcohort^33^. Genotype data from four platforms (Affymetrix 6.0, Illumina HumanHap, Illumina OmniExpress, and Illumina OncoArray) imputed to the 1000 Genomes Phase 3 version 5 reference panel were used in this study. Pre-diagnostic screening mammograms, conducted as close as possible to the blood draw date, were collected as part of a breast cancer case-control study nested within the blood subcohort. Participants have been followed up biennially through self-administered questionnaires to update exposure information and disease diagnoses. For this study, we initially included 853 women (292 cases and 561 controls) with eligible full-field digital mammograms. Among these, 383 women (143 cases and 240 controls) had available imputed genotype data and were included in the genetic analyses. Detailed descriptions of the full genotyping and quality control pipeline^34^, as well as the mammogram collection and processing procedure^21,24^, are available in previous publications. Cohort participants provided written informed consent. The study protocol was approved by the institutional review boards of the Brigham and Women’s Hospital and Harvard T.H. Chan School of Public Health, and those of participating registries as required.

### Risk factors measurement

Information on various established risk factors for breast cancer was collected for NHS II women. These factors included early life and adult body size, fat distribution, height, reproductive characteristics, and family history of breast cancer. Early life body size, WHR, height (inches), and age at menarche were reported via the baseline questionnaire in 1989. Body sizes at ages 5 and 10 years were recalled using Stunkard’s nine-level pictogram (levels 1–9: most lean to most overweight)^35^. The average of these two measurements was used to represent early life body size. For other covariates, we used the most recent information from the biennial questionnaires preceding the date of the mammogram. These covariates included: BMI, age at first birth, menopausal status, age at natural menopause, current postmenopausal hormone use, parity (number of pregnancies ≥6 months), history of benign breast disease, and family history of breast cancer. BMI (kg/m^2^) was calculated by dividing weight (kg) by the square of baseline height (m). WHRadjBMI was further calculated by regressing WHR on BMI and using the residuals from this regression.

We also assessed predicted BI-RADS density using a deep learning algorithm, which was previously developed to predict mammographic breast density from digital mammograms^31^. The algorithm categorizes breasts from a (almost entirely fatty) to d (extremely dense), matching an experienced mammographer’s evaluation (weighted *κ* for agreement with radiologists = 0.85). We coded these categories as 1, 2, 3, 4, with higher numbers indicating denser breasts. The digital mammograms used for MRS calculation were used to assess predicted BI-RADS density. Other quantitative density measures, such as absolute dense area and percent density, were not directly measured for the NHS II participants included in this study.

### Mammogram risk score measurement

The MRS is an AI-derived score capturing the texture information embedded in the whole digital mammograms, represented by millions of pixels^5,6^. It was developed utilizing 220,868 mammograms from 10,126 racially diverse, initially cancer-free women in the WashU cohort^36^, of whom 505 developed breast cancer during follow-up. Validation was performed using 150,352 mammograms from 15,885 women in EMBED, demonstrating consistently robust predictive performance (5-year AUC = 0.74)^6^. The algorithm, previously described in detail^6^, takes all standard mammogram views (CC and/or mediolateral oblique) from both breasts as input with the option of additional clinical risk factors. The outputs of the algorithm include MRS, which is a transparent weighted sum of feature coefficients, probability of 5-year breast cancer onset, and relative risk for each woman that can be used for risk calibration. For the current study, we generated an MRS for each of the 853 women by applying the algorithm to their pair of digital CC-view mammograms (one from each breast), totaling 1706 images. We used the earliest digital mammograms available for each woman.

### Genetic variants and polygenic scores for risk factors

We selected the largest available GWAS conducted among women of European ancestry for early life and adult body size, WHR, WHRadjBMI, height, age at menarche, age at first birth, age at natural menopause, number of children ever born, dense area, non-dense area, and percent density. For each risk factor, we collected lists of genetic variants reported as genome-wide significant (*P* < 5.0 × 10^−^^8^) in the original female-specific GWAS, along with their beta coefficients. When such variants were not explicitly reported, we applied PLINK’s clumping function^37^ (parameters: *P* < 5.0 × 10^−^^8^, linkage disequilibrium *r*^2^ < 0.001 within a 10 Mb window) to obtain this information. For height, for which no female-specific GWAS is known to be publicly accessible, we used genetic variant information from the largest available sex-combined GWAS. This approach was justified as no statistically significant evidence for sex differences in height genetics has been reported^38^.

To ensure these established variants were reliably imputed in the NHS II data, we included only those with matching alleles, non-ambiguous SNPs, a minimum imputation score >0.3 across all genotyping platforms, and a minor allele frequency >0.005. These selected variants were used for the PGS calculation and as IVs in causal inference analyses. Detailed information on GWAS sources and quality control of genetic variants is provided in Supplementary Table 17.

### Statistical analysis

We generated descriptive statistics for all variables. Continuous variables were described using mean and SD, while categorical variables were described using frequency and percentage. We assessed differences between higher and lower MRS groups (using the median value as the cutoff) using Student’s *t*-test or Wilcoxon rank-sum test for continuous variables and Chi-square test for categorical variables.

We first validated the association between MRS and breast cancer in NHS II using logistic regression after excluding 91 cases diagnosed within 6 months after the mammogram used for calculation. To evaluate the association between breast cancer risk factors and MRS, four main analyses were performed: (i) linear regressions of MRS on each observed risk factor to quantify their observational association without accounting for genetic predisposition; (ii) linear regressions of MRS on the PGS associated with each risk factor to evaluate the relationship between genetic predisposition to each risk factor and MRS; (iii) MR analysis via 2SLS regressions of MRS on each genetically predicted risk factor, and (iv) two-sample MR of MRS using GWAS summary statistics of each risk factor, to evaluate potential causal associations. For all analyses, we standardized MRS and all non-binary variables for easier comparison across risk factors. Binary variables included postmenopausal hormone use, history of benign breast disease, and family history of breast cancer, each categorized as “Yes” or “No.” In two-sample MR, we retained the original scale of genetic associations from the source GWAS.

For each risk factor, we calculated its weighted PGS using PLINK’s “--score” function^37^, summing the products of effect allele dosage and corresponding beta coefficient across all selected genetic variants for each woman. Prior to 2SLS regression, we assessed instrument strength by regressing each risk factor on its corresponding PGS, obtaining *F*-statistics and correlation coefficient estimates. To minimize weak instrument bias, we excluded PGS with an *F*-statistic < 5 or a correlation *P* > 0.05 from the 2SLS analysis. The 2SLS procedure involved two stages: first, regressing each risk factor on its PGS; second, using the predicted values as independent variables in a regression model with MRS as the dependent variable.

For two-sample MR, we obtained the “IVs-exposure” associations directly from the corresponding GWAS. The “IV-outcome” associations were estimated from the NHS II dataset using PLINK’s “--glm” function^37^; for our primary analysis, this was performed in the full genetic dataset (*N* = 383) with adjustment for case-control status. Our primary method was the random-effect IVW approach^39^, which assumes a zero intercept and estimates causality using random-effects meta-analysis. To validate MR model assumptions^20^ and assess the robustness of our findings, we applied complementary methods including MR-Egger regression (which detects and accounts for directional pleiotropy)^40^, weighted median (robust to up to 50% invalid instruments)^41^, weighted mode (identifies the causal effect estimate that is most consistent across all variants)^42^, and IVW excluding outlier SNPs detected using Radial MR’s iterative Cochran’s Q method^43^. We considered a causal association significant if it reached statistical significance in the IVW analysis and maintained a consistent direction across all sensitivity analyses. Following two-sample MR, we performed two additional analyses: an MR-Clust analysis to cluster genetic variants with similar causal estimates, which may reflect heterogeneous causal mechanisms^44^, and a Chi-square test on Wald ratios estimated in two-sample MR across all IVs for each risk factor to test if any of the risk factor-associated genetic variants associate with MRS.

To mitigate confounding, we employed three adjustment sets across all analyses. The crude model included age at mammogram and, where appropriate, genotyping platform and the top 10 genetic principal components. The second and third sets were additionally adjusted for menopausal status and predicted BI-RADS density, respectively. To address potential ascertainment bias arising from investigating MRS in a case-control study design that implicitly conditions on breast cancer status, we conducted all analyses using two approaches as previously recommended^30^: (1) including case-control status as an additional covariate (our primary approach to maintain sample size), and (2) restricting analyses to controls only. 2SLS and TSMR analyses were conducted using packages “ivreg”, “TwoSampleMR”, and “RadialMR” in R (v4.1.0). We used the conventional *P*-value threshold of 0.05 to define statistical significance, given the relatively limited sample size and the exploratory nature of our study.

## Supplementary information

Supplementary Information

Supplementary Data

## Data Availability

The data that support the findings of this study are available from the Nurses’ Health Studies; however, they are not publicly available. Investigators interested in using the data can request access, and feasibility will be discussed at an investigator’s meeting. Limits are not placed on scientific questions or methods, and there is no requirement for co-authorship. Additional data sharing information and policy details can be accessed at http://www.nurseshealthstudy.org/researchers. All GWAS summary statistics used in this study are publicly available.

## Code availability

Analysis scripts used to generate the results of this study are available from the corresponding author upon reasonable request.
