# Supplementary material for: Investigating the relationship between breast cancer risk factors and an AI-generated mammographic texture feature in the Nurses’ Health Study II
Source: NPJ Breast Cancer. 2025 Dec 23;12:5. doi: 10.1038/s41523-025-00870-4 (PMC12779946; doi:10.1038/s41523-025-00870-4)
Supplement: Supplementary file 1 — Supplementary Information [file 41523_2025_870_MOESM1_ESM.pdf]

Supplementary Materials

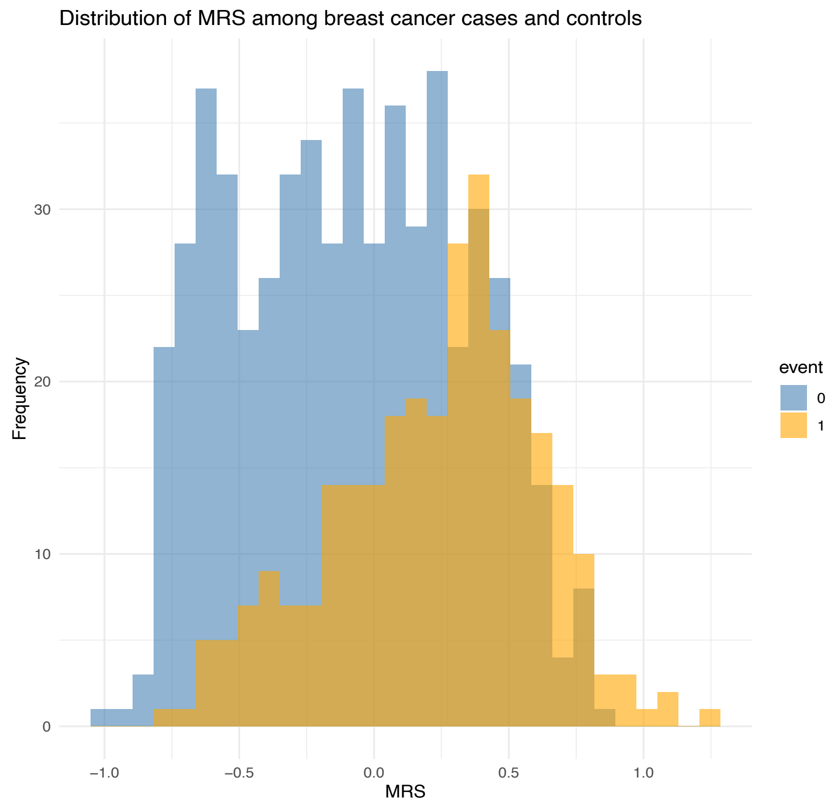

Supplementary Figure 1. Distribution of mammogram risk score (MRS) among breast cancer cases and controls in NHS II.

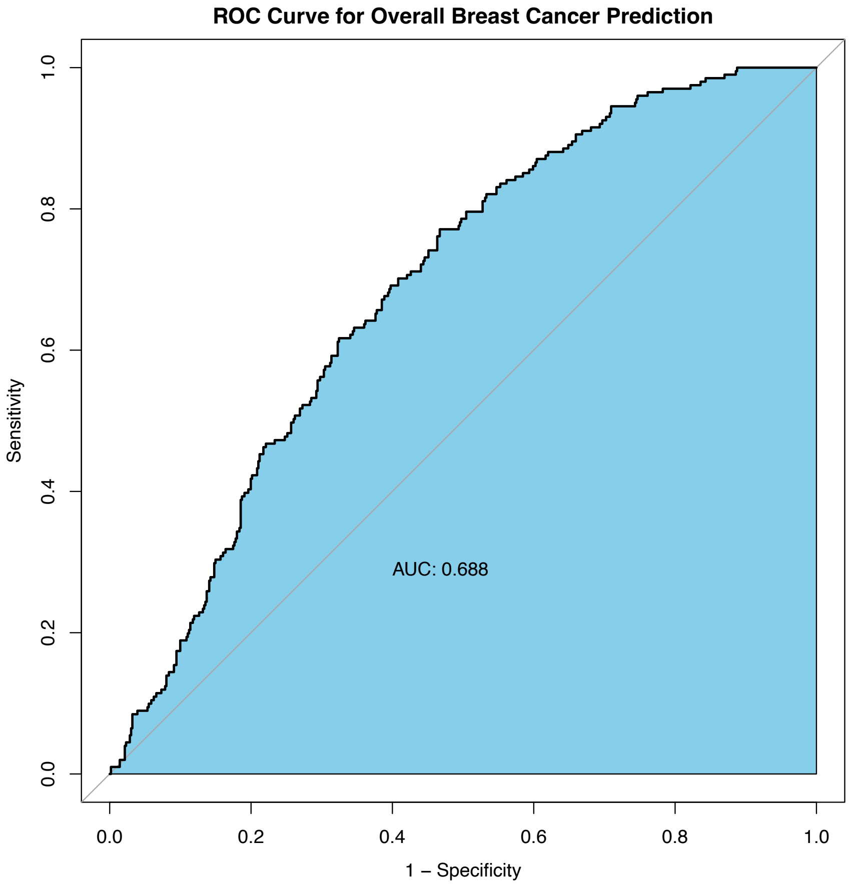

Supplementary Figure 2. Evaluating the performance of mammogram risk score in breast cancer risk prediction in NHS II.

ROC, receiver operating characteristic. AUC, area under the ROC curve.

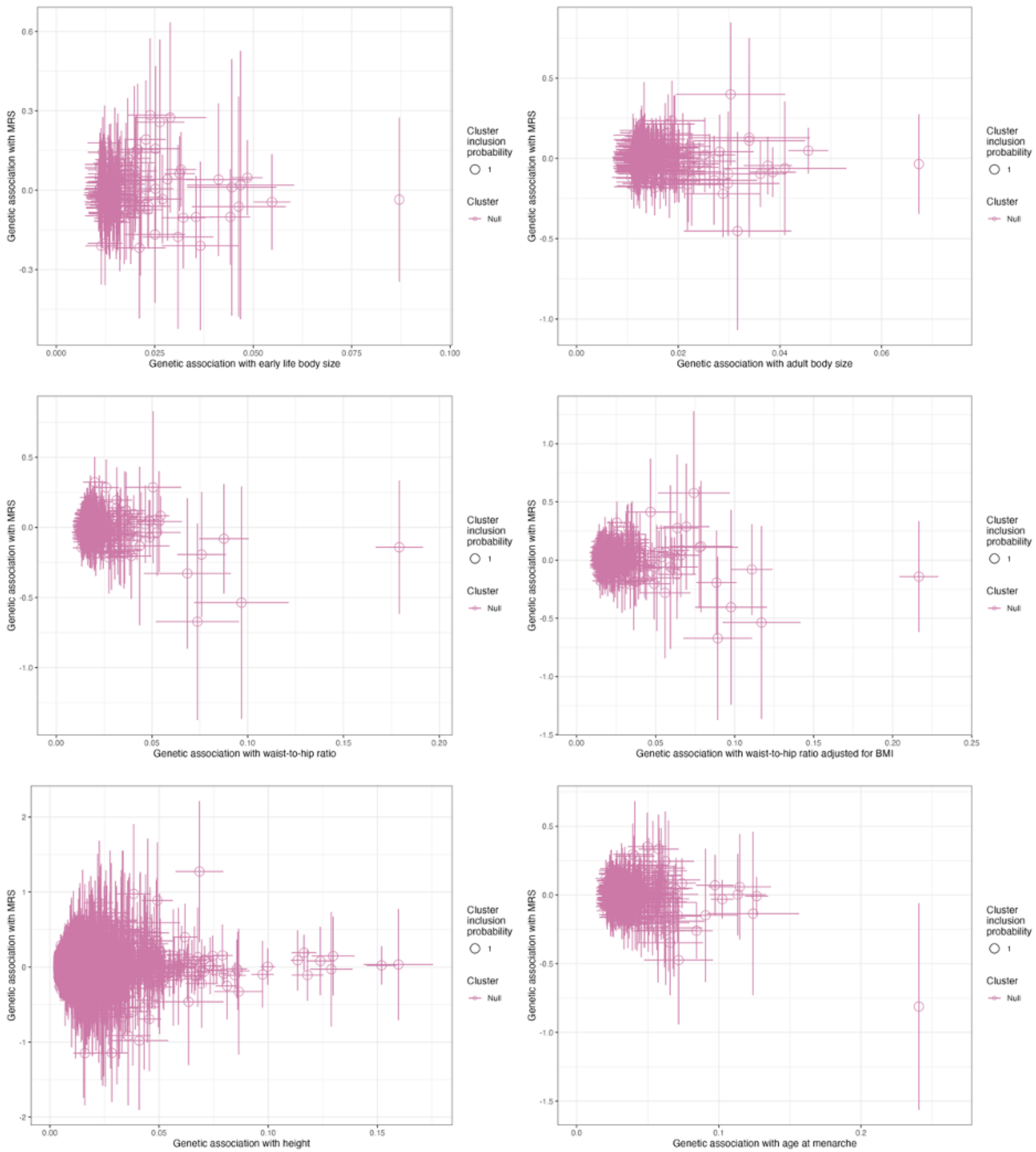

Supplementary Figure 3. Genetic associations between each risk factor and mammogram risk score (MRS). Scatter plots show results from MR-Clust analysis, with genetic association with MRS (y-axis) versus genetic association with each risk factor (x-axis). Each point represents a genetic variant, with lines indicating 95% confidence intervals.

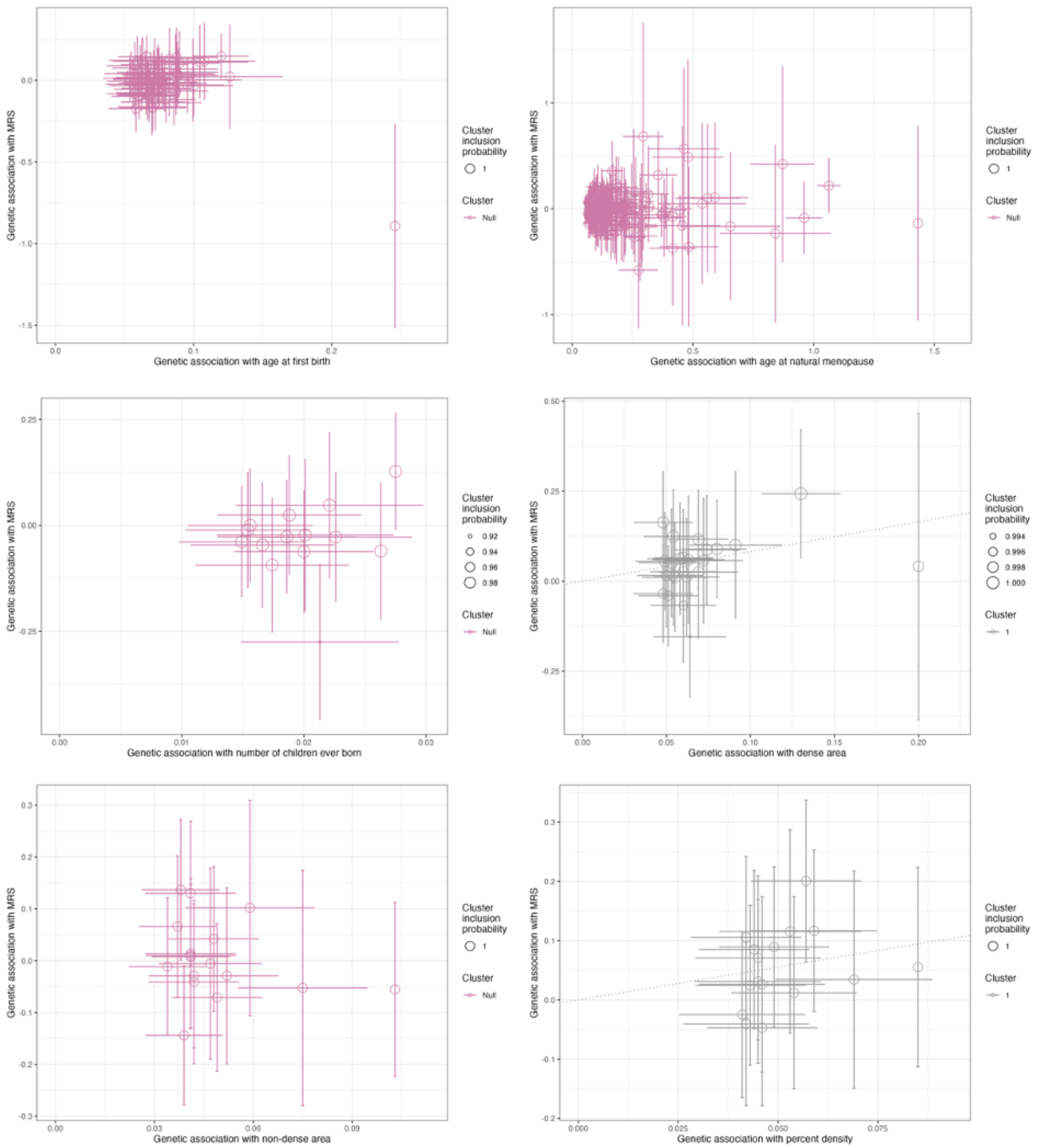

Supplementary Figure 3 (continued). Genetic associations between each risk factor and mammogram risk score (MRS). Scatter plots show results from MR-Clust analysis, with genetic association with MRS (y-axis) versus genetic association with each risk factor (x-axis). Each point represents a genetic variant, with lines indicating 95% confidence intervals.

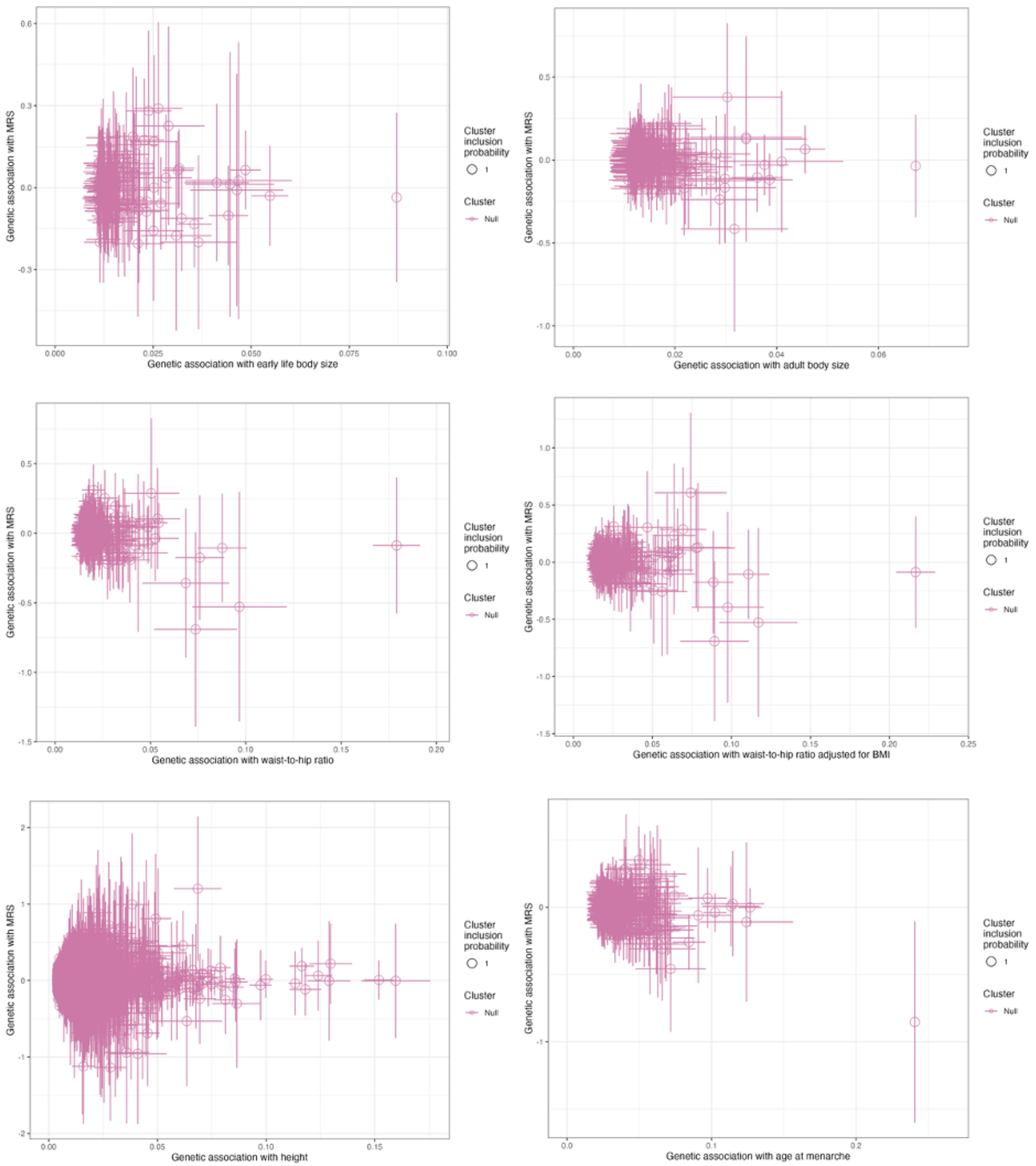

Supplementary Figure 4. Genetic associations between each risk factor and mammogram risk score (MRS) using instrumental variable-MRS associations adjusted for menopausal status. Scatter plots show results from MR-Clust analysis, with genetic association with MRS (y-axis) versus genetic association with each risk factor (x-axis). Each point represents a genetic variant, with lines indicating 95% confidence intervals.

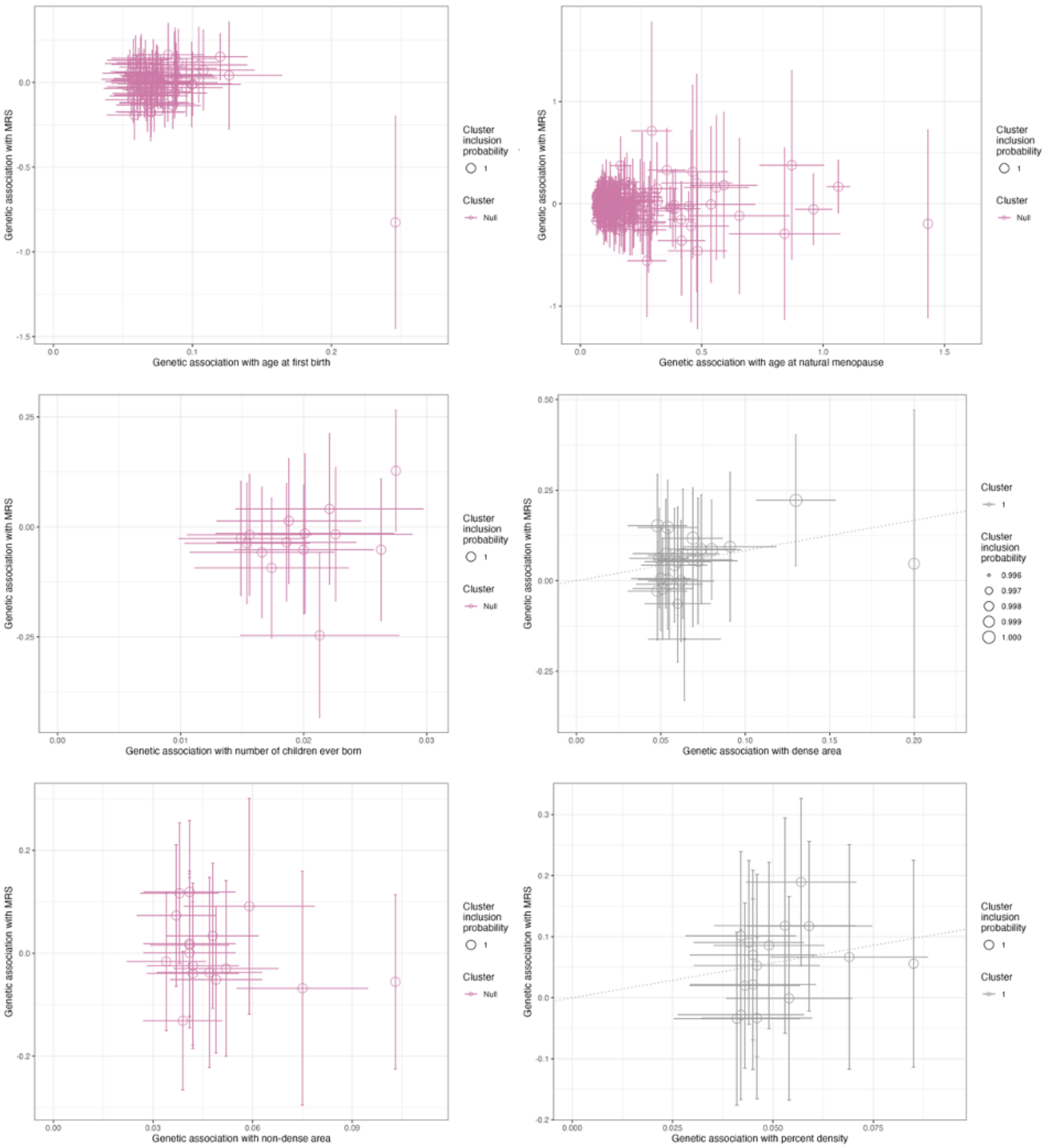

Supplementary Figure 4 (continued). Genetic associations between each risk factor and mammogram risk score (MRS) using instrumental variable-MRS associations adjusted for menopausal status. Scatter plots show results from MR-Clust analysis, with genetic association with MRS (y-axis) versus genetic association with each risk factor (x-axis). Each point represents a genetic variant, with lines indicating 95% confidence intervals.

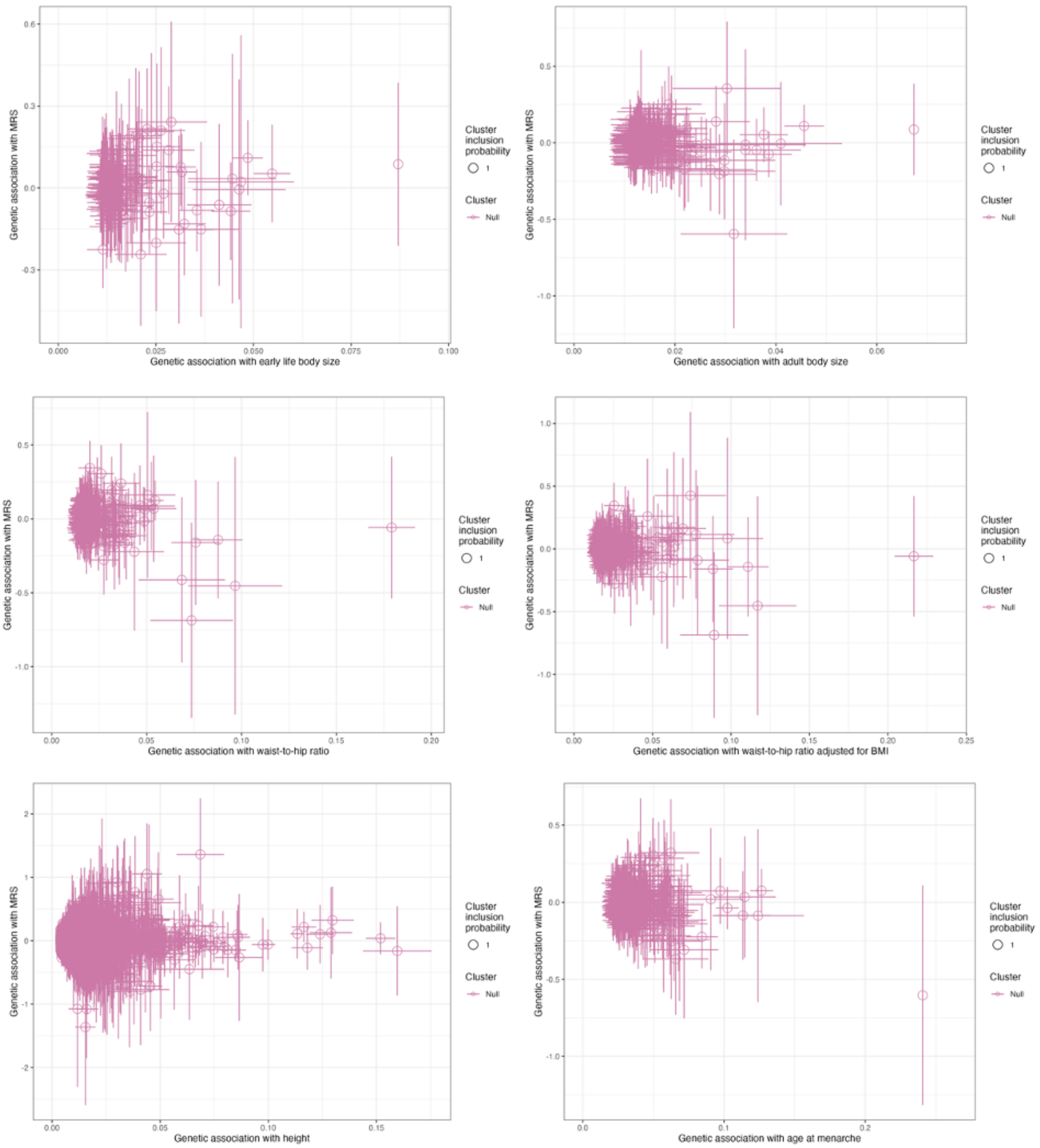

Supplementary Figure 5. Genetic associations between each risk factor and mammogram risk score (MRS) using instrumental variable-MRS associations adjusted for predicted BI-RADS density. Scatter plots show results from MR-Clust analysis, with genetic association with MRS (y-axis) versus genetic association with each risk factor (x-axis). Each point represents a genetic variant, with lines indicating 95% confidence intervals.

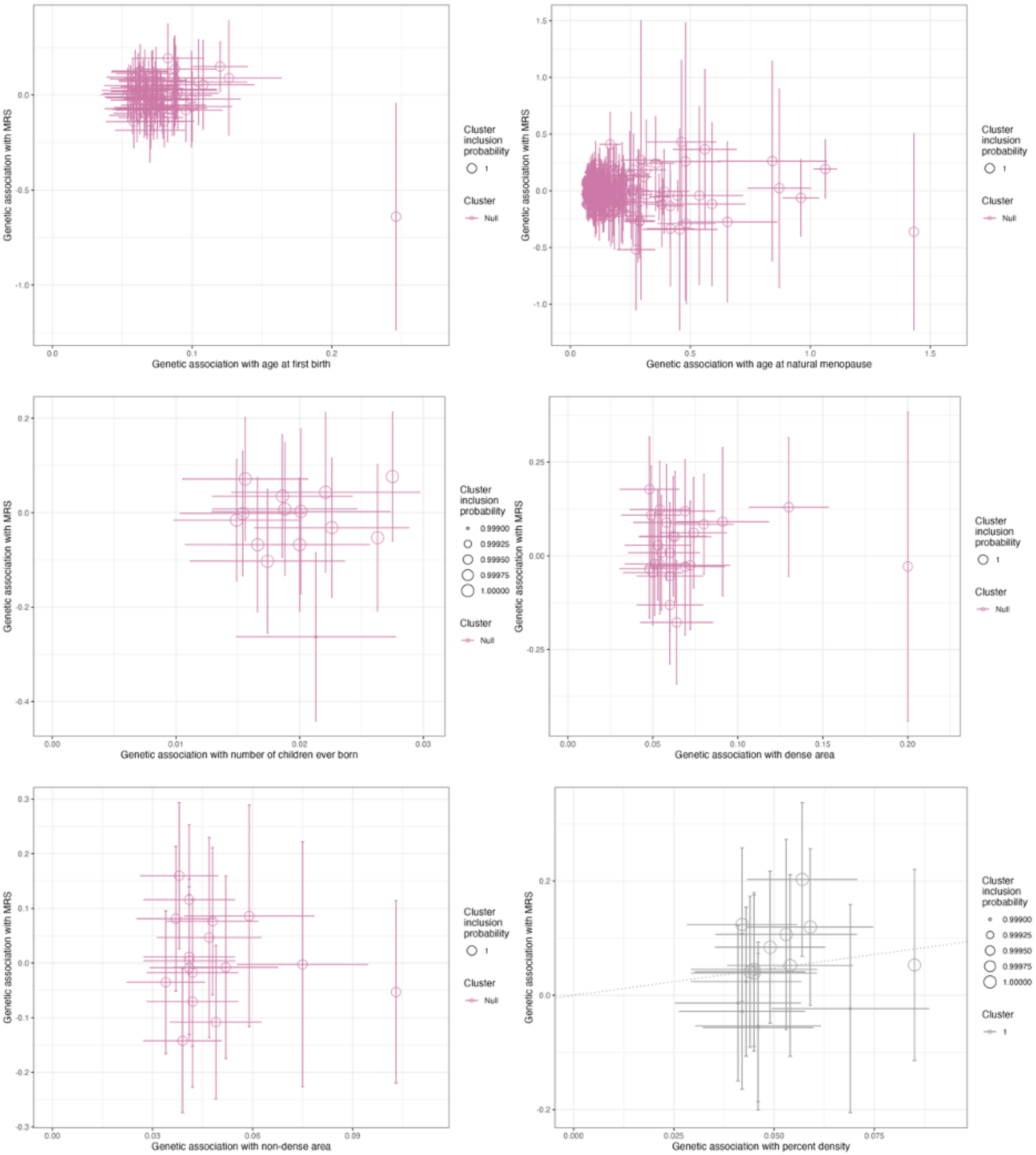

Supplementary Figure 5 (continued). Genetic associations between each risk factor and mammogram risk score (MRS) using instrumental variable-MRS associations adjusted for predicted BI-RADS density. Scatter plots show results from MR-Clust analysis, with genetic association with MRS (y-axis) versus genetic association with each risk factor (x-axis). Each point represents a genetic variant, with lines indicating 95% confidence intervals.
